# University of Louisville

**Published:** 1852-10

**Authors:** 


### THE WESTERN JOURNAL.

THIRD SERIES.—VOL. X.—NO. IV.

LOUISVILLE, OCTOBER 1, 1852.

### UNIVERSITY OF LOUISVILLE.

THE preliminary course of lectures in the medical department of the University of Louisville commences on Monday next. We take this occasion to say that Prof. Flint and Prof. Palmer are expected during the second week of the course, and that they will continue their lectures in the University throughout the winter. This statement is rendered necessary in consequence of a report which prevails in some parts of the country, that they were to remain in Louisville only a part of the winter. After the course is over here, Prof. Flint will deliver the course on Theory and Practice in the University of Buffalo.

